# Does rapid utilization of elevated nutrient availability allow eucalypts to dominate in the tropical savannas of Australia?

**DOI:** 10.1002/ece3.6168

**Published:** 2020-04-07

**Authors:** Harinandanan Paramjyothi, Brett P. Murphy, Michael J. Lawes, Natalie A. Rossiter‐Rachor, Anna E. Richards

**Affiliations:** ^1^ Research Institute for the Environment and Livelihoods Charles Darwin University Darwin NT Australia; ^2^ CSIRO Land and Water Winnellie NT Australia; ^3^ School of Life Sciences University of KwaZulu‐Natal Scottsville South Africa

**Keywords:** Escape height, noneucalypt, northern Australia, nutrient addition, tropical savanna, water addition

## Abstract

Northern Australia's savannas are among the most fire‐prone biomes on Earth and are dominated by eucalypts (*Eucalyptus* and *Corymbia* spp.). It is not clear what processes allow this group to dominate under such extreme fire frequencies and whether a superior ability to compete for nutrients and water might play a role. There is evidence that eucalypts are adapted to frequent fires; juvenile eucalypts escape the fire trap by growing rapidly in height between fires. However, non‐eucalypts are less able to escape the fire trap and tend to have stand structures strongly skewed toward suppressed juveniles. The mechanisms that drive these contrasting fire responses are not well understood. Here, we describe the results of a controlled glasshouse seedling experiment that evaluated the relative importance of nutrient and water availability in determining height growth and biomass growth of two eucalypt and one noneucalypt tree species, common in northern Australian savannas. We demonstrate that growth of eucalypt seedlings is particularly responsive to nutrient addition. Eucalypt seedlings are able to rapidly utilize soil nutrients and accumulate biomass at a much greater rate than noneucalypt seedlings. We suggest that a seasonal spike in nutrient availability creates a nutrient‐rich microsite that allows eucalypt seedlings to rapidly gain height and biomass, increasing their likelihood of establishing successfully and reaching a fire‐resistant size. Our results extend our understanding of how eucalypts dominate northern Australian savannas under extremely high fire frequencies.

## Graphical abstract

In the northern Australian savannas, eucalypts are adapted to frequent fires; juvenile eucalypts escape the fire trap by growing rapidly in height between fires. However, non‐eucalypts are less able to escape the fire trap and tend to have stand structures strongly skewed towards suppressed juveniles. The mechanisms that drive these contrasting fire responses are not well understood. We suggest that a seasonal spike in nutrient availability allows eucalypt seedlings to rapidly gain height and biomass, increasing their likelihood of reaching a fire‐resistant size and dominant recruitment.

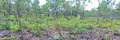

## 1 INTRODUCTION

Eucalypts (*Eucalyptus* and *Corymbia* spp.) dominate tree biomass in northern Australian savannas (60%) but account for only 28% of the tree species richness at the site level (Bowman & Prior, 2004; Lawes, Murphy, Midgley, & Russell‐Smith, 2011; Prior et al., 2006; Prior, Murphy, & Russell‐Smith, 2009; Russell‐Smith, Price, & Murphy, 2010). Given their high level of adaptations (thick bark, epicormic buds, extensive resprouting ability, etc.) to environmental stressors such as fire, the eucalypts exert strong influence over biomass dynamics in the northern Australian savannas (Burrows et al., 2002; Lawes, Richards, Dathe, & Midgley, 2011; Prior et al., 2006, 2009). In savannas where fires are frequent and/or of high severity, such as northern Australian tropical savannas, eucalypts are thought to increase in relative abundance (Bond, Cook, & Williams, 2012; Russell‐Smith, Ryan, & Durieu, 1997; Russell‐Smith et al., 2003; Williams, Cook, Gill, & Moore, 1999; Woinarski, Risler, & Kean, 2004). In northern Australian savannas, however, eucalypts co‐occur with a wide range of noneucalypt trees. The noneucalypt species includes widespread genera such as *Terminalia, Erythrophleum, Buchanania, Syzygium, and Xanthostemon* known to be suppressed by frequent fires (Fensham & Bowman, 1992; Russell‐Smith et al., 2003). A recent review suggested that the biomass dynamics of eucalypt and noneucalypt tree species are limited by different factors: canopy‐dominant eucalypts are water‐limited and typically close to carrying capacity, and the non‐eucalypts (usually found in the subcanopy) are fire‐limited and typically well below carrying capacity (Murphy, Liedloff, & Cook, 2015).

The structure and function of savanna tree communities are controlled by multiple “bottom‐up” (i.e., resource‐related) and “top‐down” (i.e., disturbance‐related) drivers. Water availability is foremost among the bottom‐up drivers and fire and herbivory are foremost among the top‐down drivers (February, Higgins, Newton, & West, 2007; Frost & Robertson, 1985; Higgins, Bond, & Trollope, 2000; Sankaran, Ratnam, & Hanan, 2004, 2008; Scholes & Archer, 1997). The roles of key top‐down drivers such as fire and herbivory in structuring savanna woody cover have been well studied (Higgins et al., 2000; Sankaran, Ratnam, & Hanan, 2004; Scholes & Archer, 1997). Similarly, the effect of a key bottom‐up driver, water, on savanna tree dynamics is well recognized (Lehmann et al., 2014; Sankaran et al., 2005). However, other bottom‐up drivers of savanna structure, especially nutrient availability, have been poorly studied.

Savanna woody cover is known to be strongly limited by water availability (Lehmann et al., 2014; Sankaran et al., 2005; Staver, Archibald, & Levin, 2011). Evidence from African savannas shows that the upper bound to tree cover is strongly correlated with mean annual rainfall (Sankaran et al., 2005). However, in much of the tropical savannas where rainfall is sufficient to support forests, the savanna occurrence is maintained by disturbances such as fire and herbivory (Bond, 2008; Cook & Goyens, 2008; Sankaran et al., 2005; Sankaran, Ratnam, & Hanan, 2008). Although there are multiple conceptual models to explain savanna tree dynamics, the fire‐mediated recruitment‐bottleneck model has attained almost universal acceptance in the global savanna literature (Bond et al., 2012; Freeman et al., 2017; Higgins et al., 2000; Prior et al., 2006; Sankaran et al., 2004). Under this model, frequent savanna fires repeatedly topkill young trees (i.e., causing death of aboveground parts), such that they must resprout at or below ground level. The loss of height that occurs each time an individual is topkilled traps it close to ground level, within the flame zone, and prevents it from becoming an adult tree, resistant to fire (Higgins et al., 2000). In the northern Australian savannas, one possible explanation for the dominance of eucalypts is that eucalypt juveniles escape the “fire trap” more easily than non‐eucalypts (Bond et al., 2012). However, the mechanisms behind this enhanced ability are unknown.

**Figure 1:**
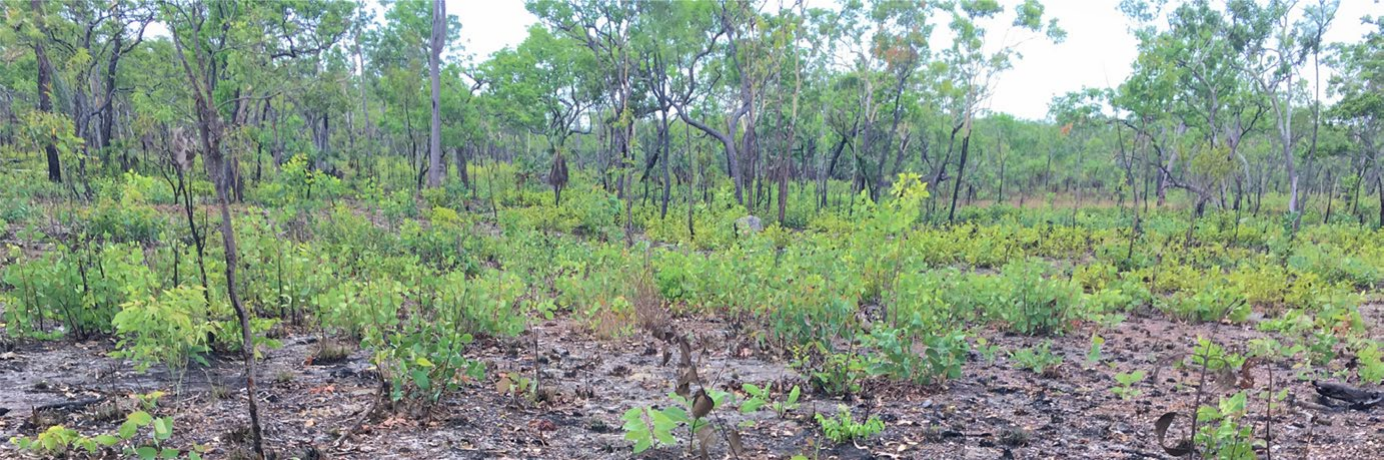
Massive regeneration of tree seedlings in the tropical savannas of northern Australia. High number of eucalypt resprout and growth observed in the early wet season after a fire event

Among other factors, seedling establishment is critical for tree species recruitment success in savanna ecosystems (Setterfield & Williams, 1996; Wilson & Bowman, 1994). Two main factors, seed availability and microsite availability, determine seedling establishment success in savannas (Harper, Williams, & Sagar, 1965; Setterfield & Williams, 1996; Setterfield, 2002). An increase in seed availability will result in increased seedling recruitment. Similarly, microsite availability (access to resources such as water and nutrients) also plays a major role in successful seedling establishment. Studies have shown that fire can positively and negatively affect seedling establishment in highly fire‐prone savannas, such as those in tropical northern Australia (Setterfield, 1996, 1997). Fire can positively influence seedling establishment by triggering seed germination and increasing nutrient availability for seedlings (Bell, Plummer, & Taylor, 1993; Lamont, Witkowski, & Enright, 1993). However, fire can also decrease seedling establishment success by reducing plant fecundity and increasing seed mortality by combusting the seeds (Setterfield, 1996, 1997). In northern Australian savannas fire could play a major role in seedling establishment success. In these savannas, studies comparing mean growth rates of juvenile plants showed varying results. Growth rates of eucalypts are typically very low, generally < 20 cm/year in height and approximately 2 mm/year in diameter at breast height (130 cm; DBH) (Cook et al., 2005; Murphy, Russell‐Smith, & Prior, 2010; Prior et al., 2006, 2009; Werner, 2005). Werner (2005) reported mean eucalypt sapling DBH growth of 2.1 mm/year, which was not significantly different from non‐eucalypts (collectively, 1.5 mm year^1^) in unburnt conditions. At these rates, saplings would require approximately 20 years to grow to fire‐resistant sizes (Bond et al., 2012), yet fires occur at annual or biennial frequencies in the region. Yet, maximum growth rates, rather than mean growth rates, may explain the escape rates of eucalypts (18%) compared with non‐eucalypts (2%) and reflect differences in the abundance of canopy trees in northern Australian savannas (Bond et al., 2012). Eucalypt escape rates (maximum growth rates) are very high after frequent intense fires, whereas noneucalypt growth is favored by long fire‐free intervals (Bond et al., 2012; Woinarski et al., 2004) (Figure 1). Species‐specific strategies such as biomass allocation and nutrient use efficiency could determine the successful recruitment of plants to the canopy layer by promoting rapid growth in order to escape from the fire trap.

Savanna soils are known to be nutrient‐poor across the tropics (Cook, 1994; Hutley & Setterfield, 2008; Lloyd et al., 2008; Murphy & Bowman, 2012). Thus, nutrient availability may be a key factor controlling tree growth in these ecosystems. In highly fire‐prone ecosystems such as savannas, nutrient cycling has been linked to postfire deposition of nutrients from burnt plant biomass (Boerner, 1982; Holt & Coventry, 1990; Rundel, 1983). Nutrients released from plant biomass can create a pulse of nutrients, particularly phosphorus, that are readily available for the plant uptake after favorable conditions such as rain events (Bodí et al., 2014; Dijkstra & Adams, 2015; Frost & Robertson, 1985; Kutiel & Naveh, 1987; Rossiter‐Rachor et al., 2009). However, studies from Australian savannas have shown that fire alone may not create an immediate spike in the availability of key nutrients (mineral nitrogen and amino acids) in the soil (Richards, Brackin, Lindsay, & Schmidt, 2012), but at the beginning of the wet season following a fire (Livesley et al., 2011; Rossiter‐Rachor et al., 2009). A rapid increase in nutrient availability following rainfall is likely to enhance plant growth (Chambers & Attiwill, 1994; Huang, Boerner, & Rebbeck, 2007; Rossiter‐Rachor et al., 2009).

In this study, we examine the responses of three canopy‐dominant savanna tree species to added water and nutrients. We quantify the effects of these treatments on plant height growth and biomass accumulation. We evaluate the hypothesis that eucalypts are able to efficiently utilize a spike in available nutrients in the soil during their juvenile stage and allocate this biomass to aboveground growth, which will help them grow taller with a higher growth rate to escape the fire trap. Additionally, we hypothesized that a similar response would be observed with additional watering.

## 2 MATERIALS AND METHODS

### 2.1 Study species

**Figure 2:**
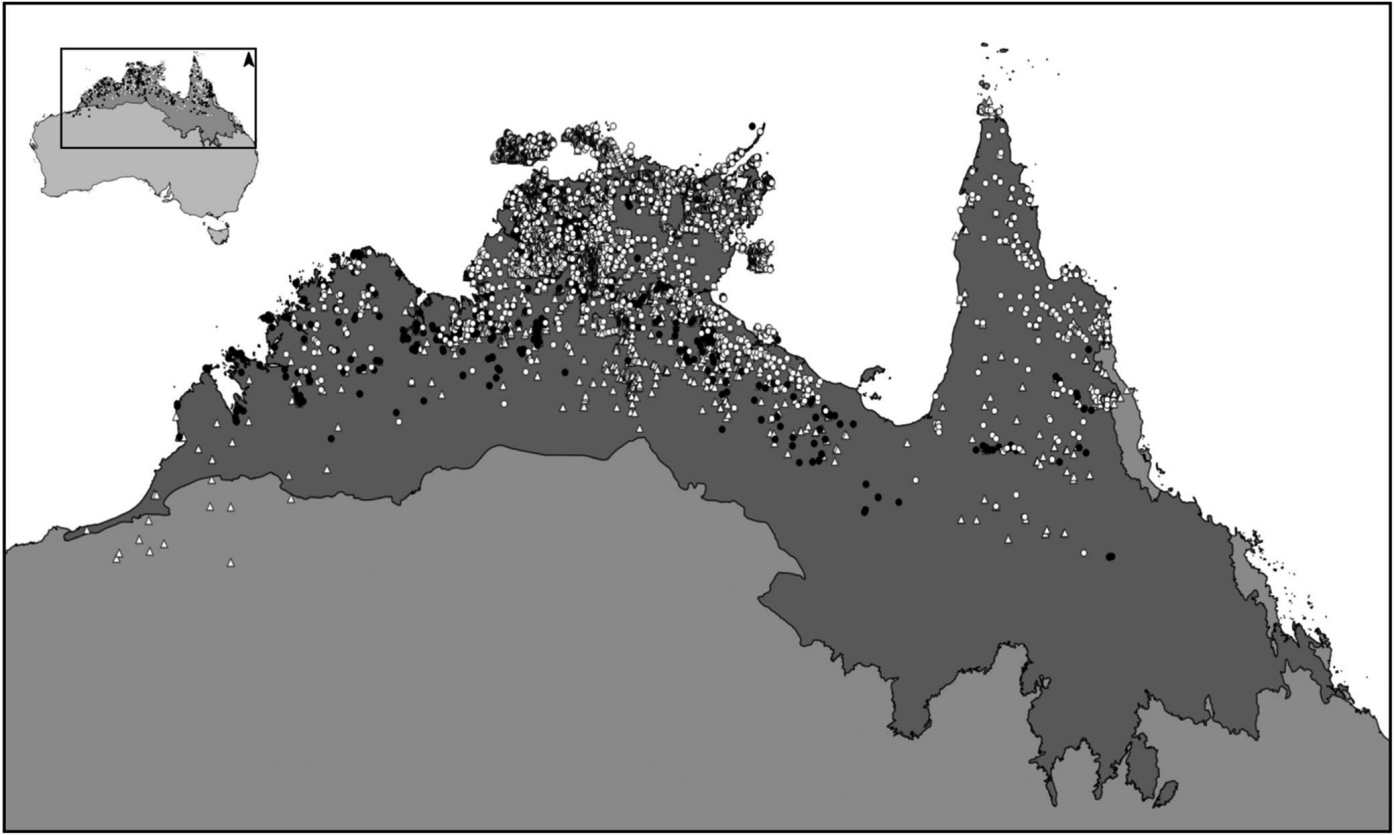
The distribution of *Eucalyptus miniata*, *Eucalyptus tetrodonta,* and *Erythrophleum chlorostachys* (dark circles, open circles, and open triangles, respectively) in the tropical savannas of northern Australia, based on collections records in Australian herbaria (Australasian Virtual Herbarium, http://avh.chah.org.au). Darker gray region in the map shows the extent of tropical savannas in Australia (Fox, Nelder, Wilson, & Bannink, 2001)

Three tree species were chosen for this study, two eucalypts (*Eucalyptus tetrodonta* and *Eucalyptus miniata*, family Myrtaceae) and one noneucalypt (*Erythrophleum chlorostachys*, family Fabaceae). These are three of the most abundant tree species throughout northern Australia's mesic savannas (mean annual rainfall > 1,000 mm) (Figure 2). Across an extensive array of vegetation monitoring sites in the mesic savannas of Kakadu, Nitmiluk, and Litchfield National Parks, near Darwin, *Eucalyptus tetrodonta* contributes, on average, 16% of total tree basal area, *Eucalyptus miniata* 16% and *Erythrophleum chlorostachys* 13% (Russell‐Smith et al., 2010).

*Eucalyptus miniata* and *Eucalyptus tetrodonta* are considered to be the most dominant trees in the northern Australian savanna woodlands (Russell‐Smith et al., 2010). Both species usually grow to 15–20 m in height but can also grow up to 30 m in favorable conditions (Setterfield, 1997). Both species regenerate mainly through vegetative reproduction (lignotubers and, for Eucalyptus tetrodonta, occasionally from root sprouts) and from seed (Lacey & Whelan, 1976; Setterfield, 1997). *Erythrophleum chlorostachys* is the dominant noneucalypt species in northern Australian savanna tree canopies, but also dominants in the sub canopy layer (Russell‐Smith et al., 2010; Setterfield, 1996, 1997). *Erythrophleum chlorostachys* grows to a maximum height of 18 m under optimum conditions and is a nitrogen‐fixing legume species (Fensham & Bowman, 1995). The study species naturally grow in the savanna woodlands of northern Australia where the soils are typical of sandy or sandy loam, extensively weathered and laterized, and climate is wet–dry tropical, with approximately 95% of the annual rainfall (~1,700 mm/year) occurring during the wet season (November–April) in Darwin (the study location) (Chen, Hutley, & Eamus, 2005).

### 2.2 Seedling establishment

The experiment was undertaken in an outdoor shade house near Darwin, Australia in 2010. Seeds from *Eucalyptus tetrodonta*, *Eucalyptus miniata,* and *Erythrophleum chlorostachys* were germinated in seedling trays (each cell 3 × 3 cm, 7 cm depth) filled with a soil mixture consisting of equal volume of washed river sand and coconut peat. *Erythrophleum chlorostachys* seeds were lightly scarified with coarse sandpaper prior to planting. Seeds were placed in shallow depressions in the soil surface and partially covered with soil. Seedlings were grown in seedling trays in a shade house under ambient temperatures and sufficient water to prevent the growth medium from drying out for 11–14 weeks. Seedlings were then transplanted into tall pots (10 cm diameter × 40 cm depth) containing natural topsoil, sourced from savanna woodland near Darwin that naturally supports the study species, mixed with approximately 20% fine sand, by volume.

### 2.3 Experimental design

Four treatments were applied to the seedlings. These treatments were designed to simulate environmental stressors faced by tree species in northern Australian savannas: moisture and nutrient limitation. A fully crossed factorial design was applied with seven replicates of each species in each treatment level. There was (1) an ambient water and no additional nutrients (Ambient), (2) ambient water and additional nutrients (N+), (3) additional water and no additional nutrients (W+), and (4) additional water and additional nutrients (W + N+) treatments. For the N + treatment, 5 g of all‐purpose fertilizer (Osmocote all‐purpose fertilizer (Scotts Australia, Everris, the Netherlands); *N* (20.9): P (0.5): K (3.8)) was added to each pot. As this is a slow‐release fertilizer (12 months of longevity), we applied a smaller amount of fertilizer per pot (1.5 g/L) than the recommended usage (5 g/L). Additional nutrients were added only once, at the beginning of the experiment. For the ambient treatment, watering occurred twice each day for 1.5 min (3 min/day), and for the W + treatment, four times per day (6 min/day). Watering occurred at a rate of 0.22 ml/cm^2^ min^−1^. Compared with the average annual rainfall of the study site (~1700 mm/year), these watering treatments were high (~2000 mm/year for ambient treatment and ~4000 mm/year for W + treatment). Extrapolation of the watering treatments to annual rainfall was done for four months (December–March) as 90% of the rainfall occurring in this period of time in Darwin region (Bureau of Meteorology, Australia). Stored water in the seedling pots was minimal due to drainage and the extra water treatment compensated this loss. We used a mist spray to water the plants so that the plants were not drenched or saturated during watering.

### 2.4 Growth metrics and statistical analysis

Height of each seedling was measured every 14 days from the beginning of the experiment. Height was measured from the cotyledon scar to the growing tip. The experiment was terminated after six months, all seedlings were harvested, and biomass of each seedling was separated into leaves, stems, roots, and lignotubers. The mass of each component was measured after ovendrying the samples at 80°C for 48 hrs.

We examined four seedling response variables, each measured at the end of the experiment: height; root:shoot ratio; total biomass; and belowground biomass. All response variables were log‐transformed prior to analysis to ensure normality. Generalized linear modeling was used to investigate the effects of the experimental treatments on each seedling response variable (R package glm2; Marschner, 2014). The three species were analyzed separately.

The best model of each seedling response variable was selected using Akaike's information criterion (AIC) (Table S1 & S2). We began the model selection with a saturated model, containing two binary predictor variables (W + treatment, N + treatment, and their interaction) and then iteratively removed predictor variables, searching for the model with the lowest AIC. If an individual predictor did not add sufficient explanatory power relative to its contribution to model complexity, as defined by AIC, it was dropped from the model. All analyses were performed in RStudio version 3.4.1 (RStudio, 2015) and the R package “ggplot2” (Wickham, 2016) used for diagrams.

## 3 RESULTS

### 3.1 Seedling height

**Figure 3:**
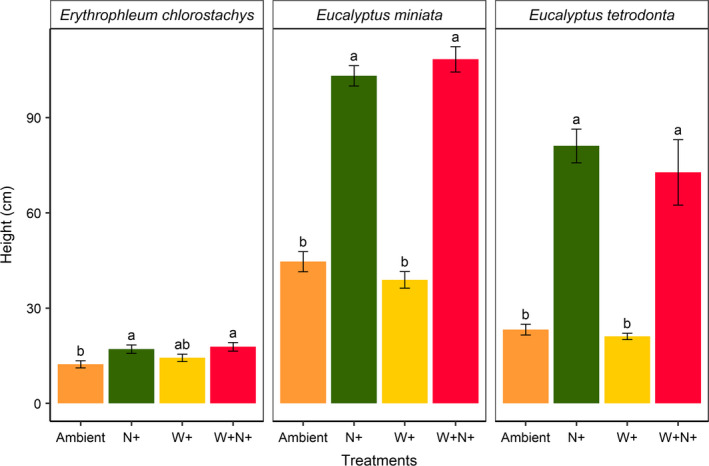
Final height of seedlings under various nutrient and water treatments. Treatment labels “Ambient,” “N+,” “W+,” and “W + N+” indicate ambient water and nutrients; nutrient addition; water addition; and addition of both water and nutrients, respectively. Letters above the error bars indicate significant differences between treatments (*p* < .05). Errors bars represent standard error of the mean

For all species, we observed a significant increase in height with the addition of nutrients, relative to ambient nutrient levels (Figure 3). The effect was greatest in both eucalypt species; *Eucalyptus miniata* was 131% taller (*p* < .001), relative to ambient, and *Eucalyptus tetrodonta* was 248% taller, while *Erythrophleum chlorostachys* was 38% taller (*p* < .001). Addition of water had no effect on the height of seedlings of any species.

### 3.2 Total biomass

**Figure 4:**
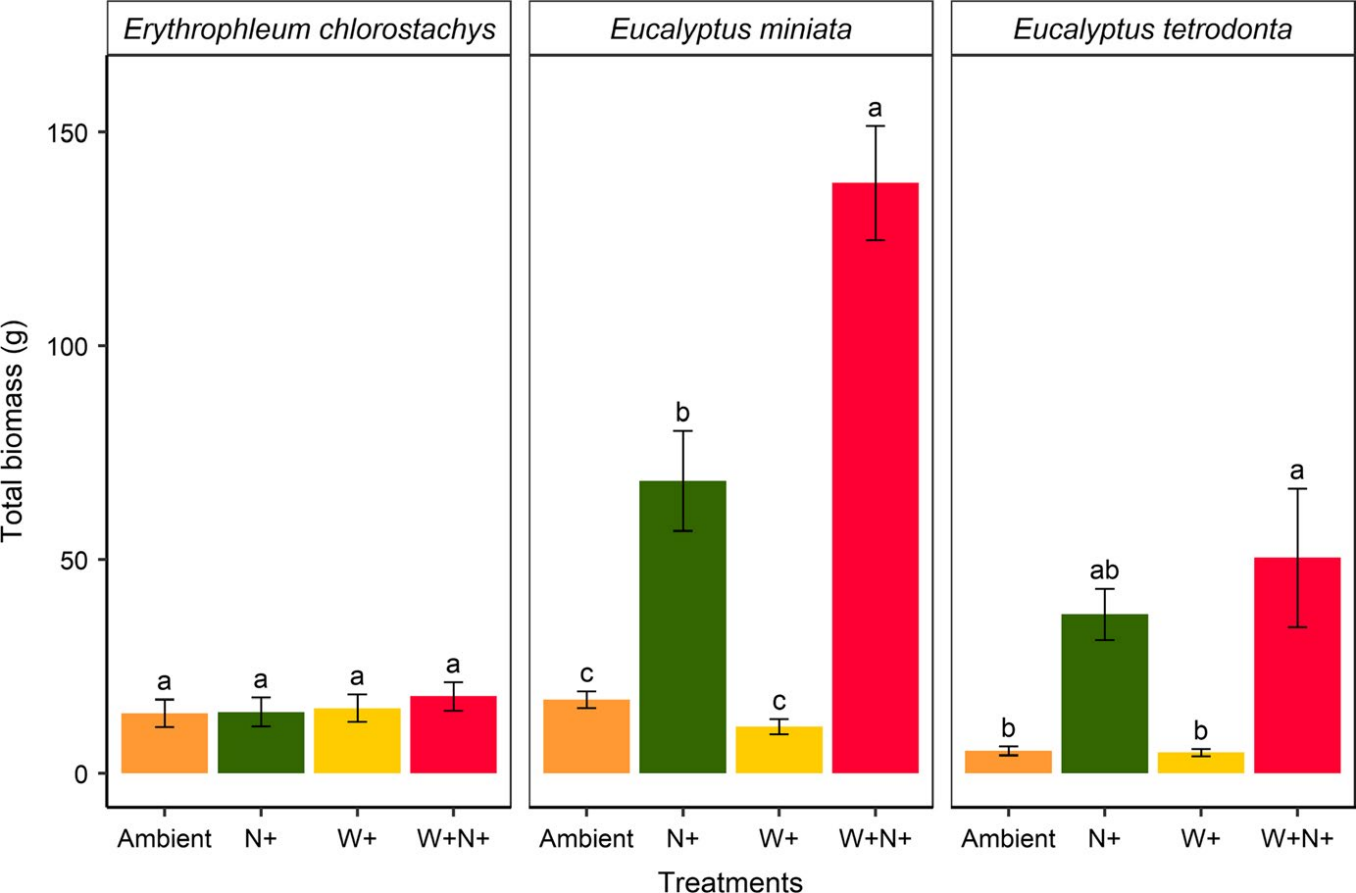
Final total biomass of seedlings under various nutrient and water treatments. Treatment labels “Ambient,” “N+,”, “W+,” and “W+N+” indicate ambient water and nutrients; nutrient addition; water addition; and addition of both water and nutrients, respectively. Letters above the error bars indicate significant differences between treatments (*p* < .05). Errors bars represent standard error of the mean

*Eucalyptus miniata* showed a large increase in total biomass with the addition of nutrients (*p* < .01), relative to ambient. Addition of water also slightly increased *Eucalyptus miniata* total biomass (*p* < .01), while interaction of water and nutrient addition (W + N+), resulted in a significant increase of 121g total biomass compared with ambient (Figure 4). Similarly, *Eucalyptus tetradonta* was highly responsive to nutrient addition with a significant increase in total biomass (*p* < .01) compared with the ambient treatment. However, *Erythrophleum chlorostachys* total biomass was unresponsive to water and nutrient additions.

### 3.3 Belowground biomass

**Figure 5:**
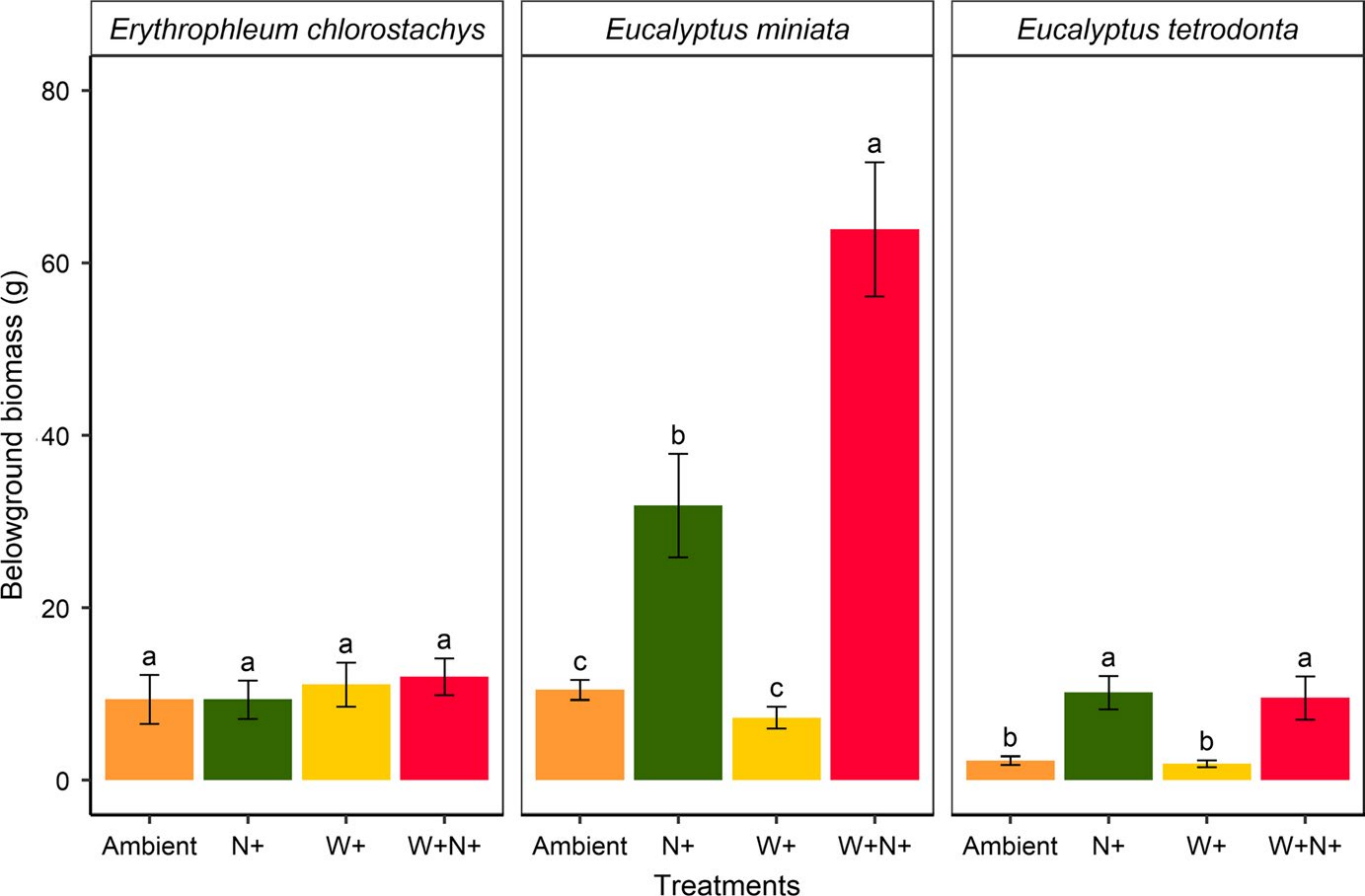
Final belowground biomass of seedlings under various nutrient and water treatments. Treatment labels “Ambient,” “N+,” “W+,” and “W+N+” indicate ambient water and nutrients; nutrient addition; water addition; and addition of both water and nutrients, respectively. Letters above the error bars indicate significant differences between treatments (*p* < .05). Errors bars represent standard error of the mean

There was a significant interaction between the additional water and nutrient treatments in relation to belowground biomass of *Eucalyptus miniata* (biomass was 508% higher than ambient; *p* < .01). However, only nutrient addition had a significant positive effect on *Eucalyptus tetrodonta* belowground biomass (*p* < .05) (Figure 5). *Erythrophleum chlorostachys* showed no significant response to nutrient or water addition in terms of belowground biomass. We found higher belowground biomass allocation in *Erythrophleum chlorostachys* compared with *Eucalyptus miniata* and *Eucalyptus tetrodonta* under the absence of nutrients. Among the eucalypts, *Eucalyptus miniata* had higher belowground biomass than *Eucalyptus tetrodonta*, primarily reflecting the presence of a lignotuber in *Eucalyptus miniata.*

### 3.4 Root:shoot ratio and biomass allocation

**Figure 6:**
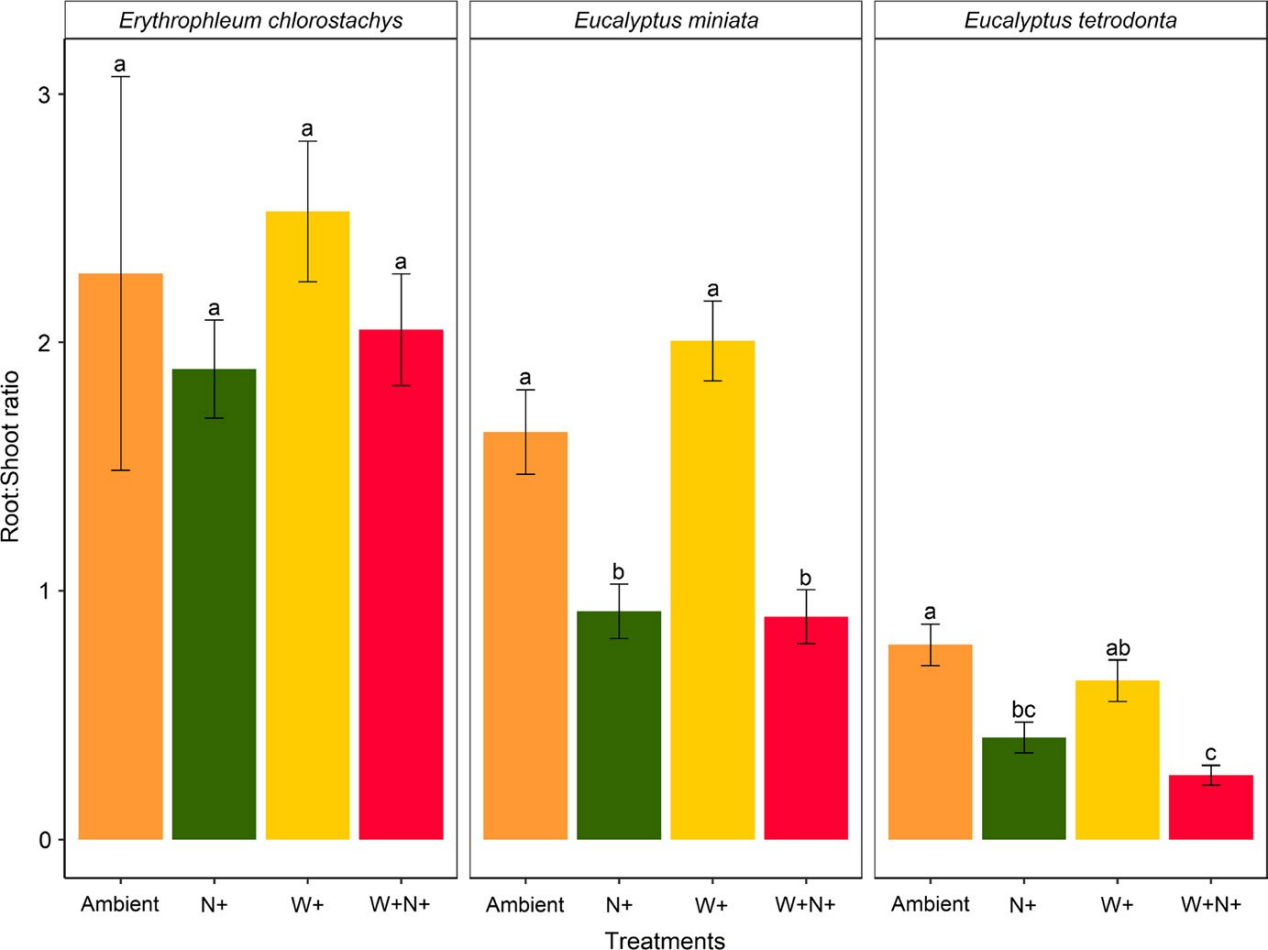
Final root:shoot ratio of seedlings under various nutrient and water treatments. Treatment labels “Ambient,” “N+,” “W+,” and “W+N+” indicate ambient water and nutrients; nutrient addition; water addition; and addition of both water and nutrients, respectively. Letters above the error bars indicate significant differences between treatments (*p* < .05). Errors bars represent standard error of the mean

In both eucalypt species, addition of nutrients resulted in a decrease in root:shoot ratio. (*Eucalyptus miniata*, *p* < .001; *Eucalyptus tetrodonta*, *p* < .05) (Figure 6). *Erythrophleum chlorostachys*, which had a higher root:shoot ratio than the two eucalypts, did not show a significant response to either water or nutrient treatments.

## 4 DISCUSSION

In northern Australia, the dominant savanna trees, eucalypts, can successfully recruit into adult size classes under regimes of very frequent fires. Conversely, the recruitment of non‐eucalypts to the canopy tends to be negatively affected by such fire regimes (Bond et al., 2012; Murphy et al., 2015; Freeman et al., 2017). The mechanistic basis of the remarkable ability of higher eucalypts establishment and escape the so‐called “fire trap” has not been identified (Bond, 2012; Setterfield, 2002). Our results suggest that high rates of recruitment of eucalypts into the canopy under frequent fire may be related to their ability to utilize abundant soil nutrients in the early wet season when there is less resource competition from both non‐eucalypts and the grass layer that have been impacted by fire. Eucalypt seedlings are able to rapidly utilize soil nutrients and accumulate biomass at a greater rate than the seedlings of an important codominant tree species of Australia's mesic savannas, *Erythrophleum chlorostachys*. Whether this relative inability to utilize nutrients is characteristic of a wider range of the noneucalypt species is unclear.

Northern Australian savanna soils tend to be nutrient‐poor, at least in part due to frequent fires depleting nutrient stocks over time, as savanna fires lead to the loss of macro‐ and micronutrients to the atmosphere during combustion of biomass (Cook, 1994; Hutley & Setterfield, 2008). However, it is also likely that there is a seasonal spike in the availability of soil nutrients due to the release of plant‐available nutrients stored in biomass burnt during the fire and released when soil moisture content increases with the wet season rains (Bodí et al., 2014; Dijkstra & Adams, 2015; Frost & Robertson, 1985; Rossiter‐Rachor et al., 2009). In savannas, postfire increases in soil nutrient concentrations are thought to be largely confined to the surface layers of the soil and are maintained for up to a month after fire, and then decline rapidly (Frost & Robertson, 1985). This spike in available nutrients may lead to the creation of a nutrient‐rich microsite for the seeds and seedlings in the soil (Lamont et al., 1993; Setterfield, 1996, 1997). Evidence from northern Australian savannas suggests a clear pulse of available nitrate and ammonium in the soil at the beginning of wet season rains after a fire (Rossiter‐Rachor et al., 2009). In other fire‐prone, nutrient‐poor plant communities, such as the Mediterranean heathlands of southwestern Australia, many woody plant species have life histories finely tuned to take advantage of the postfire nutrient pulse—typically with fire‐cued germination of soil‐stored seed or fire‐cued release of canopy‐stored seed (Bell et al., 1993). Our results suggest that a similar phenomenon may exist in northern Australian savannas, resulting in higher rates of seed germination and seedling establishment. That is, seedlings of the two dominant tree species of northern Australia's mesic savannas (*Eucalyptus tetrodonta* and *Eucalyptus miniata*) are highly responsive to nutrient addition and capitalize on the seasonal increase in available soil nutrients during the early wet/growing season when there is a lack of competition for nutrients from non‐eucalypts (if they have been impacted by dry season fires) and the grass layer (which is yet to re‐establish after fire). This may explain why low‐intensity fires appear to promote the growth of Eucalypts in field conditions (Freeman, Murphy, Richards, Vesk, & Cook, 2018; Prior et al., 2006; Werner, 2005; Werner & Prior, 2013). However, the unresponsiveness of *Erythrophleum chlorostachys* seedlings to nutrient addition may be explained by their nitrogen‐fixing ability, which would lesson their reliance on soil‐stored nutrients for growth, such that carbon availability may be the main growth‐limiting factor in this species (Coskun, Britto, & Kronzucker, 2016). *Erythrophleum chlorostachys* is considered to be a slow‐growing species in these savannas and, in some cases, may never escape the fire trap. However, they are able to persist through fire events probably because of their thicker bark and successfully reproduce within the flame zone (unlike Eucalypts) (Freeman et al., 2018; Lawes, Richards, et al., 2011).

Eucalypts play a central role in the biomass dynamics of northern Australian savannas, yet the role of nutrients in controlling biomass dynamics in these savannas has received little attention. Our results highlight the importance of differences in growth responses of different savanna tree species to nutrients in their juvenile stage, although we cannot clarify which nutrients (e.g., nitrogen and phosphorus) are driving this response. Although enhanced soil nutrient concentrations led to a significant increase in eucalypt biomass, both total and belowground biomass, our results show a significant decrease in root: shoot ratio in eucalypt species with the addition of nutrients. In spite of the fact that no significant differences in treatments, *Erythrophleum chlorostachys* showed a higher root:shoot ratio compared with eucalypts in all treatments. In our results, both eucalypts allocated biomass in the aboveground plant parts to grow taller at a higher rate compared with the noneucalypt. This trade‐off between above‐ and belowground biomass accumulation in each study species can be explained as a growth strategy, whereby eucalypts allocate most biomass aboveground to establish from seedling stage and escape the flame zone while *Erythrophleum chlorostachys* stores more biomass in their roots to regenerate after the fire event, similar to other savanna trees around the world (Chapin III, Schulze, & Mooney, 1990; Hoffmann & Franco, 2003; Hoffmann, Orthen, & Franco, 2004; Schutz, Bond, & Cramer, 2009; Tomlinson et al., 2012; Wigley, Cramer, & Bond, 2009). Our preliminary analysis of species‐specific responses with/without treatments showed a higher growth rate in both eucalypts compared with *Erythrophleum chlorostachys*. Additionally, the noneucalypt exhibited a preferred belowground allocation strategy whereas the eucalypts showed high growth rates and grew taller (Table S2; Figure S1 & S2). Such a dichotomy would be expected based on the “lanky” (e.g., eucalypts) versus. “corky” (e.g., midstory non‐eucalypts) typology of Dantas and Pausas (2013). Whether other northern Australian savanna non‐eucalypts store more biomass belowground than aboveground is unclear. The total unresponsiveness of the study species to water addition could be because these savanna tree seedlings are not limited primarily by water (Wilson & Bowman, 1994) and that the ambient water treatment provided sufficient moisture for seedling growth without any stress to the study individuals.

## 5 CONCLUSION

We have investigated the responses of three woody species in the northern Australian savannas, two eucalypts (*Eucalytptus miniata* and *Eucalyptus tetrodonta*) and one noneucalypt (*Erythrophleum chlorostachys*) to nutrient and water addition, focusing on patterns of growth and biomass allocation. We recognize that many other biotic and abiotic factors, including tree and grass competition, could influence the responses of woody plant dynamics in savannas. Nevertheless, our results contribute to the fundamental understanding of the processes promoting the high rates of recruitment of eucalypts, and their dominance of the canopy, specifically the potential importance of a seasonal spike in nutrient availability that creates a nutrient‐rich microsite for the seedlings at the onset of wet season rains. Whether other non‐eucalypts in the northern Australian savannas respond similarly to *Erythrophleum chlorostachys* and what specific nutrients help eucalypts to dominate in these savannas is unclear. Although this study provides insights into the dominance of eucalypts, we were not able to show the potential determinants of *Erythrophleum chlorostachys* and similar non‐eucalypts abundance in these savannas. Additionally, we recognize that this study is limited to seedlings in controlled environments, and more study is required to test whether these responses can be extended to field situations and to larger saplings and mature trees. However, our results strongly suggest that the successful recruitment of eucalypts into the canopy may be related to their ability to utilize seasonally abundant nutrients under the absence of noneucalypt and grass competition, which helps them to establish dominance and rapidly escape the fire trap.

## CONFLICT OF INTEREST

The authors declare that they have no conflict of interest.

## AUTHOR CONTRIBUTIONS

All authors contributed to the preparation of manuscript. The writing was led by Harinandanan Paramjyothi with the assistance of all other authors. Anna Richards and Michael Lawes designed the study and collected the data. Harinandanan Paramjyothi analyzed the data with the help from Brett Murphy and Anna Richards. All authors reviewed and approved the final manuscript.

## ETHICAL APPROVAL

The work described here did not require ethics approval.

## Data Availability

Data associated with this paper are available in Dryad, https://doi.org/10.5061/dryad.x69p8czf2
