# Supplementary figures and images for: Does rapid utilization of elevated nutrient availability allow eucalypts to dominate in the tropical savannas of Australia?
Source: Ecol Evol. 2020 Apr 7;10(9):4021–30. doi: 10.1002/ece3.6168 (PMC7244804; doi:10.1002/ece3.6168)

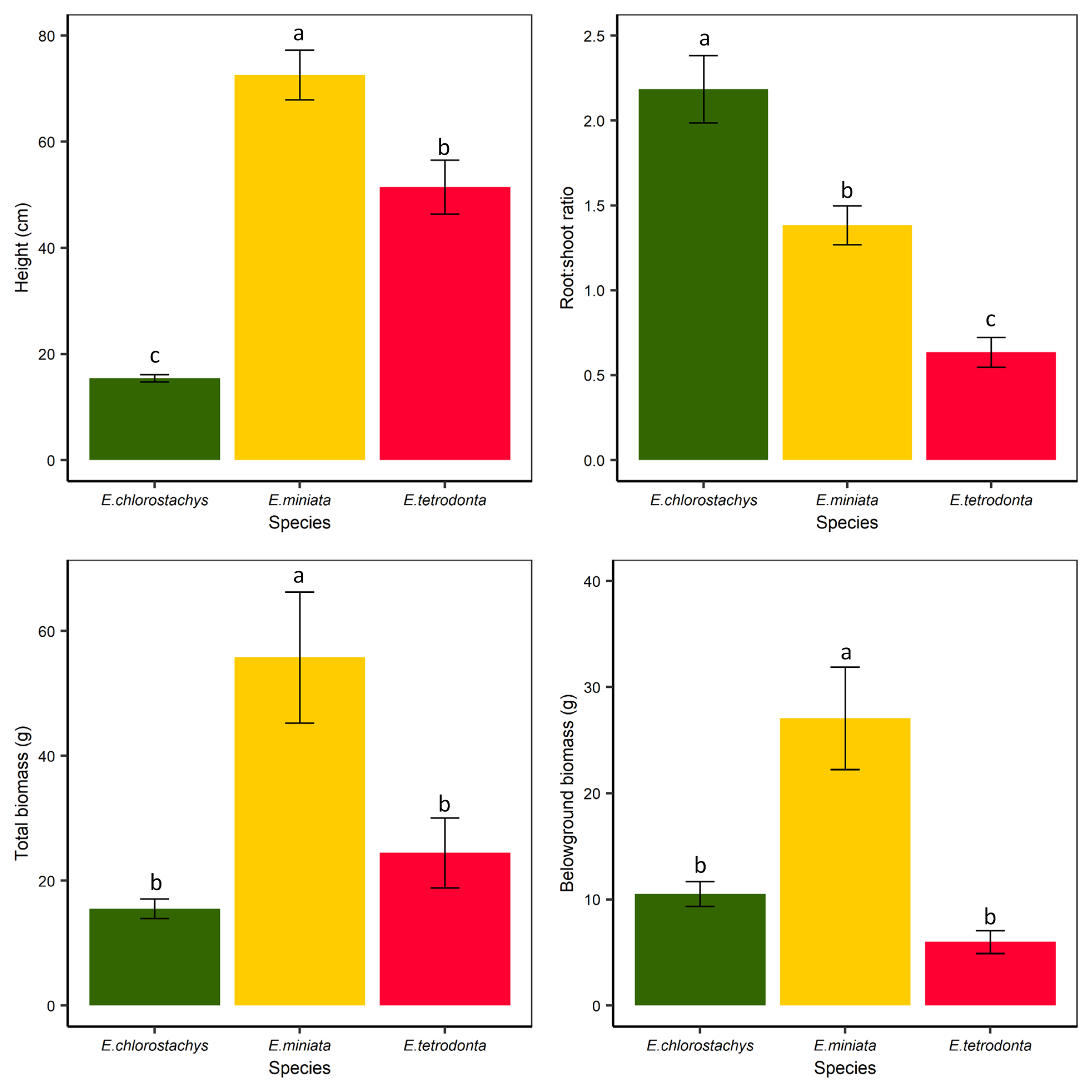

Supplement: Supplementary file 1 [file ECE3-10-4021-s001.tif]

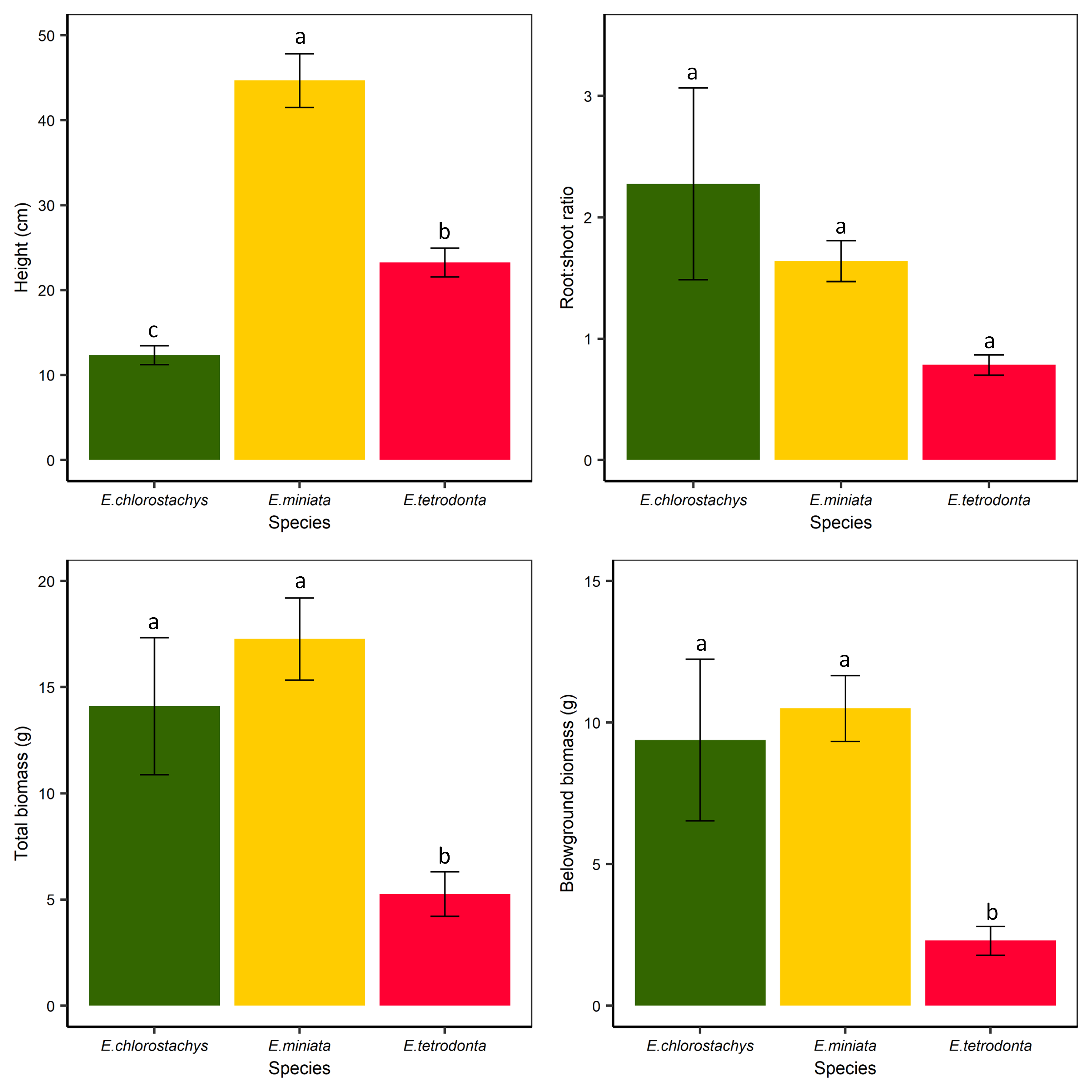

Supplement: Supplementary file 2 [file ECE3-10-4021-s002.tif]
