# Supplementary material for: Does rapid utilization of elevated nutrient availability allow eucalypts to dominate in the tropical savannas of Australia?
Source: Ecol Evol. 2020 Apr 7;10(9):4021–30. doi: 10.1002/ece3.6168 (PMC7244804; doi:10.1002/ece3.6168)
Supplement: Supplementary file 3 [file ECE3-10-4021-s003.docx]

Table S1. Model summaries for each seedling response variable, for each study species. N+, W+ and N+W+ represents nutrient addition, water addition and interaction of nutrient and water additions respectively. Best models are in bolded font.

| **Response variable** | **Species** | **Models** | **AIC** | **R2** | **p** |
| --- | --- | --- | --- | --- | --- |
| Height | E. miniata | **N+** | **127** | **0.87** | **<0.05** |
|  |  | W+ | 237 | -0.18 | 0.85 |
|  |  | N+ + W+ | 128 | 0.86 | <0.05 |
|  | E. tetrodonta | **N+** | **162** | **0.7** | **<0.05** |
|  |  | W+ | 219 | -0.01 | 0.45 |
|  |  | N+ + W+ | 162 | 0.7 | <0.05 |
|  | E. chlorostachys | **N+** | **25** | **0.17** | **<0.05** |
|  |  | W+ | 35 | 0.006 | 0.25 |
|  |  | N+ + W+ | 25 | 0.18 | <0.05 |
| Total biomass | E. miniata | **N+** | **44** | **0.79** | **<0.05** |
|  |  | W+ | 87 | -0.04 | 0.92 |
|  |  | N+ + W+ | 46 | 0.78 | <0.05 |
|  | E. tetrodonta | **N+** | **93** | **0.32** | **<0.05** |
|  |  | W+ | 105 | -0.02 | 0.5 |
|  |  | N+ + W+ | 95 | 31 | <0.05 |
|  | E. chlorostachys | N+ | 62 | -0.04 | 0.82 |
|  |  | W+ | 62 | -0.01 | 0.43 |
|  |  | N+ + W+ | 63 | -0.05 | 0.71 |
| Belowground biomass | E. miniata | **N+** | **45** | **0.71** | **<0.05** |
|  |  | W+ | 79 | -0.37 | 0.79 |
|  |  | N+ + W+ | 46 | 0.71 | <0.05 |
|  | E. tetrodonta | **N+** | **75** | **0.43** | **<0.05** |
|  |  | W+ | 92 | -0.03 | 0.6 |
|  |  | N+ + W+ | 77 | 0.42 | <0.05 |
|  | E. chlorostachys | N+ | 67 | -0.04 | 0.89 |
|  |  | W+ | 66 | -0.0001 | 0.33 |
|  |  | N+ + W+ | 68 | -0.04 | 0.62 |
| Root:shoot ratio | E. miniata | **N+** | **15** | **0.58** | **<0.05** |
|  |  | W+ | 40 | -0.02 | 0.46 |
|  |  | N+ + W+ | 17 | 0.58 | <0.05 |
|  | E. tetrodonta | **N+** | **54** | **0.14** | **<0.05** |
|  |  | W+ | 59 | -0.04 | 0.85 |
|  |  | N+ + W+ | 56 | 0.11 | 0.09 |
|  | E. chlorostachys | N+ | 34 | -0.02 | 0.5 |
|  |  | W+ | 33 | 0.01 | 0.27 |
|  |  | N+ + W+ | 35 | -0.01 | 0.44 |

Table S2. Model summaries of interspecific responses. N+, W+ and Sps represents nutrient addition, water addition and species identity respectively. The top panel is model summaries of species responses across treatments. The bottom panel represents the model summaries of species responses only for the control treatment individuals. Best models are in bolded font.

| **Response variable** | **Models** | **AIC** | **R2** | **p** |
| --- | --- | --- | --- | --- |
| Height | N+ | 343 | 0.22 | <0.05 |
|  | W+ | 382 | -0.006 | 0.74 |
|  | N+ + W+ | 345 | 0.21 | <0.05 |
|  | Sps | 245 | 0.58 | <0.05 |
|  | Sps + W+ | 247 | 0.58 | <0.05 |
|  | **Sps+ N+** | **106** | **0.83** | **<0.05** |
|  | Sps + N+ + W+ | 108 | 0.83 | <0.05 |
| Total Biomass | N+ | 383 | 0.3 | <0.05 |
|  | W+ | 414 | -0.008 | 0.55 |
|  | N+ + W+ | 384 | 0.31 | <0.05 |
|  | Sps | 400 | 0.16 | <0.05 |
|  | Sps+ W+ | 401 | 0.16 | <0.05 |
|  | **Sps + N+** | **359** | **0.5** | **<0.05** |
|  | Sps+ N+ + W+ | 360 | 0.5 | <0.05 |
| Belowground Biomass | N+ | 321 | 0.2 | <0.05 |
|  | W+ | 340 | -0.008 | 0.56 |
|  | N+ + W+ | 322 | 0.2 | <0.05 |
|  | Sps | 312 | 0.3 | <0.05 |
|  | Sps + W+ | 313 | 0.3 | <0.05 |
|  | **Sps + N+** | **282** | **0.52** | **<0.05** |
|  | Sps + N+ + W+ | 282 | 0.52 | <0.05 |
| Root:Shoot ratio | N+ | 78 | 0.06 | <0.05 |
|  | W+ | 83 | -0.004 | 0.4 |
|  | N+ + W+ | 79 | 0.06 | <0.05 |
|  | Sps | 25 | 0.51 | <0.05 |
|  | Sps + W+ | 26 | 0.51 | <0.05 |
|  | **Sps + N+** | **13** | **0.59** | **<0.05** |
|  | Sps + N+ + W+ | 13 | 0.59 | <0.05 |
| Control individuals | | | | |
| Height | **Sps** | **16** | **0.59** | **<0.05** |
| Total Biomass | **Sps** | **32** | **0.54** | **<0.05** |
| Belowground Biomass | **Sps** | **37** | **0.62** | **<0.05** |
| Root:Shoot ratio | **Sps** | **29** | **0.4** | **<0.05** |
